# Effect of Enterprise Social Media on Employee Creativity: Social Exchange Theory Perspective

**DOI:** 10.3389/fpsyg.2021.812490

**Published:** 2022-01-21

**Authors:** Zhiwei Wang, Mahri Hangeldiyeva, Asad Ali, Mengmeng Guo

**Affiliations:** ^1^Department of Economics and Management, Nanjing Agricultural University, Nanjing, China; ^2^College of Liberal Arts, Technological University of the Philippines, Manila, Philippines; ^3^School of Economics and Management, Zhejiang Sci-Tech University, Hangzhou, China; ^4^Department of Electrical Engineering, Foundation University, Islamabad, Pakistan; ^5^College of Literature and Journalism, Sichuan University, Chengdu, China

**Keywords:** enterprise social media usage, leader-member exchange, support for innovation, social media usage frequency, employee creativity

## Abstract

This study applied an artifact-centric view to investigate the consequences of enterprise social media usage. It investigates how enterprise social media usage influences employee creativity. A moderated mediation model is developed based on social exchange theory. The empirical sample of 238 employees is used to test the proposed model. Results of the empirical analysis performed using PROCESS macro of SPSS indicate that enterprise social media usage positively impacts employee creativity via the mediating mechanisms (i.e., leader-member exchange and support for innovation). Furthermore, social media usage frequency negatively moderates this impact of enterprise social media usage on employee creativity via leader-member exchange. Interestingly, the empirical analysis reveals that the impact of enterprise social media usage frequency strengthens the indirect effect that enterprise social media usage has on employee creativity via perceived support for innovation.

## Introduction

Social media is extensively used in organizations around the world (Liang et al., 2020). Social media is used with the perception that it will increase the efficiency of interaction among organizational members (Chen et al., 2019), facilitate knowledge exchange (Wei et al., 2020), and increase customer satisfaction (Casper Ferm and Thaichon, 2021). Considering associated benefits, a majority of companies from the group of Fortune 500 have adopted social media (Ali et al., 2020). Yet, besides its benefits, studies have noted that social media use by employees can have negative consequences. For example, Salo et al. (2018) and Cao and Yu (2019) noted that social media use is related to a decrease in employee performance. Furthermore, Bao et al. (2020) found that social media use can result in fatigue and frustration. Thus, this backlash of social media for employees suggests further development of research and theory to understand the consequences enterprise social media use might have for the development of organizations and the performance of its employees.

Even though many organizations are ambivalent in social media adoption. However, the Covid19 pandemic has changed the work structures, especially how knowledge workers perform their jobs (Trougakos et al., 2020). Due to the Covid19 pandemic, approximately 50% of the knowledge workers now work remotely from home (Kuruzovich et al., 2021). In addition, this study noted the possibility that remote work or work from home may last for more years to come. This suggests that the implications of enterprise social media usage for employee performance are critical, especially in the current remote work situation. Thus, a study to investigate the impact of enterprise social media usage and employee performance is important to increase our understanding of how social media may affect employee job performance.

A literature review indicates that enterprise social media has mixed positive and negative impacts on employee performance outcomes. For instance, Ali et al. (2021a) found a positive relationship between enterprise social media usage and innovation performance. Meanwhile, contrasting to above, Yu et al. (2018) noted that when employees excessively utilize social media, this brings negative consequences to their performance. The inconsistencies in the empirical studies might be because studies generally focus on knowledge management and social capital which limits the scope of mechanism that might link social media with job related outcomes (Pitafi et al., 2018; Ali and Bahadur, 2019). Therefore, considering previous inconsistencies, recent studies have suggested theoretically elaborate and empirically test how enterprise social media brings benefits to employees. Especially, studies have asked called to investigate how social media can enhance employee creativity (Sigala and Chalkiti, 2015; Wang et al., 2021).

To answer the above calls, this study argues that a theoretical integration of information systems literature and organizational behavior literature might bring novel insights into how enterprise social media is linked with employee creativity. From the perspective of information systems, we consider enterprise social media as a technological artifact (Cao et al., 2021). From the organizational behavior perspective, we draw on social exchange theory to propose a mechanism that links enterprise social media usage with employee creativity. Social exchange theory is used because it explains social and psychological processes that underlie employees’ job-related behavior and outcomes. Based on the social exchange theory, we identify social and psychological processes mechanisms to explain the relationship between our technological artifact (enterprise social media) with employee creativity and social media externalities as a moderator in these relationships. Thus, different from previous studies linking social media with employee performance through knowledge-centric mechanisms (Liu et al., 2014; Chen et al., 2019; Nisar et al., 2019; Ali et al., 2020), we build a theoretical model that includes both information systems perspective and organizational behavior perspective.

Our study makes some valuable contributions to the social media and creativity literature. First, this study answers the calls to conduct more theory-driven research to explain the consequences of enterprise social media usage. In doing so, we use social exchange theory to investigate how enterprise social media usage by employees is linked to their creativity. Particularly, we identify LMX and SFI as key mechanisms that link social media use with employee creativity. Second, we investigate enterprise social media usage frequency as an important moderator that strengthens the impact of enterprise social media usage on employee creativity via a social exchange mechanism. Third, this study contributes to the existing literature on information systems by investigating social exchange mechanisms differently than previous studies which are predominantly focused on knowledge-oriented mechanisms linking social media with employee job outcomes.

### Theory and Hypotheses

#### Theoretical Foundation: Social Exchange Theory

Social exchange theory (Blau, 1964) describes the behavior during long and short-term relationships where tangible (i.e., money) and intangible resources (i.e., social support) are exchanged. Social exchange theory in particular describes the social exchange of tangibles and intangibles as a social exchange process. This theory has been applied to illuminate numerous circumstances and behaviors. For instance, social exchange theory is used to explain the behavior of customers (Casper Ferm and Thaichon, 2021), employee job performance (Kuruzovich et al., 2021), inter-organizational exchanges, and trust (Lioukas and Reuer, 2015), and the relationship between leader and peers (Miao et al., 2014). At its core, social exchange theory argues that social exchanges are made among actors over the time of their relationship creating trust, affect, and loyalty (Blau, 1964; Quade et al., 2020). Social exchange is a reciprocal process between interaction partners (Adams, 1963). For example, a transaction of tangible or intangible resources would be reciprocated by the receiving exchange partner at some time in the future. Although researchers have studied creativity from a variety of theoretical perspectives (Santos et al., 2015; Tu et al., 2018; Klasmeier and Rowold, 2020; Ali et al., 2021b), due to the social nature of creativity (Amabile, 1996; Perry-Smith and Shalley, 2003; Grosser et al., 2014), social exchange theory provides a useful lens to investigate social exchanges processes among organizational members using enterprise social media that eventually enhance employees creativity.

**FIGURE 1:**
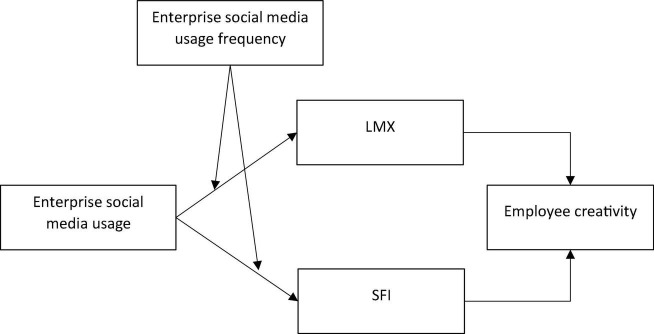
Proposed theoretical model.

LMX and SFI represent two important constructs that compute aspects of social exchange. LMX, as suggested in the literature is related to the exchange of resources between employees and their leaders (Graen and Uhl-Bien, 1995). On the other hand, SFI involves an exchange between peers (Ali et al., 2021b). This exchange relationship resultantly develops perceived support for innovation by the employees (Anderson and West, 1998). Although, LMX and SFI are theoretically distinct, yet, these exchange relationships dominantly provide the foundation in social exchange theory to capture the processes that affect employees’ behavior and job outcomes. Moreover, although, previously, studies have noted the importance of these factors, yet, in the context of social media, these factors have not been studied in any detail, nor LMX and SFI are collectively used in a study. This study proposes that LMX and SFI are important mechanisms that link enterprise social media usage with employee creativity. Figure 1 presents the proposed theoretical model of this study.

### Hypotheses Development

#### Social Exchange and Employee Creativity

The social model of creativity proposes (Perry-Smith and Shalley, 2003) that creativity is a social process that involves exchange among individuals. Accordingly, creativity is one of the key consequences of social exchange processes (Cao and Ali, 2018). As such, LMX and SFI have positive implications for employee creativity. When individual employees work in the conditions of a high level of LMX and SFI, they feel a sense of indebtedness and obligation to the sources of treatment. Accordingly to the social exchange norm of reciprocity (Adams, 1963), employees feel motivated to repay the resources in a form that is appreciated by the resource providers. When the exchange partner is a leader or supervisor (LMX), employees feel confident in meeting job requirements by taking indicative and sharing ideas to meet leader expectations. When the exchange partner is a co-worker, employees feel confident that their ideas are valued, and their co-workers are supportive of their creative ideas. Together, LMX and SFI employees demonstrate a high level of commitment toward creatively solving problems and generating solutions to defuse situations. More precisely, LMX and SFI may enhance employee creativity by creating efficiencies through social exchange. For instance, on one hand, LMX may enhance employee creativity because knowledge exchange with leader, gaining valuable resources, and political clout of managers is important for an employee to take a risk and adopt creative ways of performing their tasks (Zhao, 2015; Hu et al., 2018; Tu et al., 2018). On the other hand, SFI may contribute to employee creativity again by providing cognitive resources and support for creative actions (Ali et al., 2021b). This is because creativity needs the exchange of ideas and support for co-workers in order to improve ideas and select a creative idea which is also useful (Shin and Zhou, 2003). In summary, LMX and SFI are might contribute to employee creativity by providing them the necessary resources through social exchanges with leaders and peers which are argued to enhance employee confidence and ability to generate creativity (Shin and Zhou, 2003, 2007).

#### Social Exchange and Employee Creativity

The social model of creativity suggests that creativity is a process of social exchange among members (Perry-Smith and Shalley, 2014). Accordingly, we identified LMX and SFI as key exchange factors influencing employee creativity. For instance, when employees receive a favorable LMX and SFI, they may feel a sense of indebting to the sources of LMX and SFI. In line with the sense of reciprocity, employees try to reply to the sources in a favorable way which may be valued by the sources. When the exchange partner of an employee is the leader (i.e., LMX) reciprocal behavior may include sharing creative ideas and taking a risk, and generating creative solutions to the problems (Zhou et al., 2018). Meanwhile, when the exchange partner is a peer or co-worker (i.e., SFI), an appropriate reciprocal behavior could be shared and discuss ideas, knowledge, and perspectives to identify novel and effective ideas and increase creativity (Ali et al., 2021b). Together, LMX and SFI, therefore, increase employee creativity as frequent exchange processes with others in the organization provide valuable resources that help employees to develop novel and useful ideas.

#### The Mediating Role of Social Exchange Processes

We believe that social exchange will play a role in mediating the relationship between employee creativity and the use of social media in the workplace. Enterprise social media usage is may bring additional benefits to exchange processes by easing the interaction and communication among members of an organization, therefore, positively affecting the relationships among individuals. Moreover, research has indicated that social media use by employees creates an understanding of knowledge, personality, and attitude among individual employees (Ali et al., 2019). This reduces the uncertainties regarding work-related behaviors and social related behaviors of employees and leaders. Such understanding is likely to make it easy for employees to fully interpret the LMX relationship with the leader. Moreover, the LMX relationship is characterized by mutual trust and respect, which are easily observed when interactions are not ambiguous and interaction partners can easily interpret. In addition, an important characteristic of enterprise social media usage is that it enables collaborative conditions (Cardon and Marshall, 2015). Since, enterprise social media usage creates situations in which employees discuss, share, and support the work of each other to complete the task efficiently (Majchrzak et al., 2013), it may create a perception that other co-workers provide support for innovation and thus an employee is more confident to generate creative ideas discuss it with other employees using social media to identify original and effective ideas. Additionally, enterprise social media provides a tool that encourages and eases the social exchange relationships (i.e., LMX and SIP) among individuals to support each other as they work toward attaining creativity (Ali et al., 2019). For the above reasons, we draw on social exchange theory to identify that enterprise social media usage by employees and leaders affects their social exchange relationships in a way that these exchange relationships provide support to individuals to exchange resources and support to generate creative solutions.

In sum, based on the social exchange theory, we establish how LMX and SIP are promoted through the use of enterprise social media by individuals in an organization to enhance their creativity.

Hypothesis 1: LMX mediates the relationship between enterprise social media usage and employee creativity.

Hypothesis 2: SFI mediates the relationship between enterprise social media usage and employee creativity.

#### Enterprise Social Media Usage Frequency and LMX

In this section, this study proposes that through a reduction in the quality of LMX, the frequency of company social media usage will have a detrimental impact on the link between enterprise social media usage and employee creativity. Enterprise social media usage contributes to enhancing the exchange relationships by facilitating interaction and communication among employees (Zhang et al., 2019). A review of the literature reveals that using enterprise social media influences performance outcomes depending upon the usage frequency by employees. Such as, Cao and Yu (2019) noted that when employees extensively use social media at work this is negatively related to their performance. Managers with more frequent access to enterprise social media that is managers more frequently interact with subordinates using social media may lead to interactional uncertainty among leader and sub-ordinates compared with face-to-face interaction, resulting in low quality exchange relationships. This maybe particularly important in the context when leaders extensively use social media because in such situation exchange resources such as feelings of positive effect, warmth, and gestures may not be transferred to the followers. If such exchange resources cannot be transferred in social exchange through LMX interaction in enterprise social media and there is less opportunity for face-to-face interaction due to extensive social media use. Enterprise social media usage is likely to hinder the development and maintenance of LMX resulting in a low level of employee creativity. Therefore, we hypothesize that.

Hypothesis 3: Enterprise social media usage frequency will moderate the indirect relationship between enterprise social media usage and employee creativity via LMX such that the indirect relationship becomes weak when leaders more frequently use enterprise social media.

#### Enterprise Social Media Usage Frequency and SFI

In this section, this study states that the association between enterprise social media usage and employee creativity via SFI will also be hampered by the frequency of enterprise social media usage. Enterprise social media can contribute to enhancing social exchange relationships by improving employee perceptions that co-workers are supportive in generating innovative solutions. There is theoretical and empirical support that SFI leads to increased creativity by creating favorable and supportive work conditions (Ali et al., 2021b). Additionally, research has suggested that creativity is a social process (Perry-Smith and Shalley, 2003), this social side is of creativity is supported by enterprise social media usage (Ali et al., 2020). Yet, extensive use of enterprise social media might decrease the quality of interaction. That is when employees more use enterprise social media to interact with each other, this decreases face-to-face interaction. By using extensive enterprise social media employees are unable to know other communication signals and social support which is possible in physical interaction, thereby decreasing the perceptions about support for innovation provided by co-workers. In other words, to the extent to which employees feel supported by co-workers through the use of enterprise social media, they will have a lower level of perceived SFI when they are more frequently using enterprise social media to interact with each other, resulting in a lower level of creativity.

Hypothesis 4: Enterprise social media usage frequency will moderate the indirect relationship between enterprise social media usage and employee creativity via SFI such that the indirect relationship becomes weak when employees more frequently use enterprise social media.

### Research Methods

#### Field Settings and Data Collection

We used a survey method to test the hypotheses of this study. This methodology allows capturing the essence of the fieldwork, therefore it is considered an appropriate method to study our variables (Liu et al., 2014). Data were collected from employees working in companies in developing region. Following previous studies (Cao and Ali, 2018; Pitafi et al., 2018; Khan et al., 2019; Cao et al., 2021), data were collected from employees working in multiple companies.

At first, 21 companies were identified using academic-industrial networking located in developing region. Then, we made telephone calls to HR managers and sent them the invitation email explaining the purpose of this research and requesting their voluntary participation. The HR managers were guaranteed the confidentiality of the data collected from their employees. Through this process, managers of seven companies agreed to participate in the survey. Second, we conducted informal interviews of the HR managers to confirm that employees use enterprise social media to communicate and interact with each other.

After the initial process, HR managers are considered an appropriate source of authentic information about organizational policies and practices. HR department supported us to distribute the questionnaire to the employees at two points in time. In phase one, we distributed the questionnaire to 391 employees. In response, we received 261 responses. One month later in phase two, we distributed the questionnaire to the respondents of the first phase and asked them to rate their creativity. In response, we received 243 responses. After removing incomplete responses, we have 238 useful responses from the employees. The demographic characteristics of the respondents indicate that there are 123 (51.7%) female respondents and 115 (48.3%), male respondents, most of the respondents’ age (79.8%) is below 35 years, and most of the respondents have a bachelor degree (53.4%).

### Measures

We used all scales validated from previous studies. In the survey, all items were measured using 7 points Likert scale (1 = strongly disagree to 7 = strongly agree). ***Enterprise social media*** is measured using six items adapted from Ou and Davison (2011). Previous studies (Cai et al., 2018; Pitafi et al., 2018) have validated this measure. The social exchange processes were measured using validated measures from the literature. Particularly, ***LXM*** was assessed using four items. These items are adopted from the study of Graen and Uhl-Bien (1995). For ***SFI*,** three items were adapted from Anderson and West (1998). We asked employees to provide the ***frequency of using enterprise social media*** by the number of times they use enterprise social media to interact, communicate, and exchange knowledge with each other. Finally, a self-reported measure of ***employee creativity*** was adopted from Farmer et al. (2003). This is a validated approach in previous studies to measure the creativity of employees using self-ratings (Ding et al., 2019).

Since all survey measures are self-reported, this could bring the possibility of common method bias (Podsakoff and Organ, 1986). Therefore, to avoid such potential effect of common method bias on the results of this study, we adopted two methods. First, we measured dependent variables at different times, such time-lagged surveys could mitigate the potential arousal of common method bias (Podsakoff et al., 2003). Second, to investigate the likelihood of common technique bias, we used Harman’s single factor test (Harman, 1976). Results indicate that no single factor accounted for a majority of the variance. Indeed, the first factor accounted for 23% of the total variance, and the Eigen value of 4 factors was greater than 1 cumulating 64% of the total variance. Thus, the test results suggest that common method bias is not a serious issue in our study.

## Results

We performed data analysis using SPSS. Particularly, we used PROCESS macro a plugin to test mediation hypotheses and moderated mediation hypotheses of this study. This tool is commonly used for exploratory research conducted by previous studies (Eissa and Lester, 2017; Islam et al., 2020; Bahadur and Ali, 2021). We conducted the data analysis in two phases. First, Model 4 of PROCESS macro is to test the mediation effect. Thus, we used it to test the mediation hypotheses. Second, we used Model 7 of PROCESS macro to test the moderated mediation hypotheses.

Table 1 correlation matrix shows the reliabilities, correlations among key variables of the study. This table provides initial evidence of the support for our model.

**TABLE 1:** Correlation matrix.

Variables	Mean	*SD*	1	2	3	4	5	6	7	8	9
1. Age	1.48	0.50	–								
2. Gender	1.77	0.83	–0.08	–							
3. Education	3.39	0.62	0.09	0.07	–						
4. UE	2.38	0.57	–0.05	0.02	–0.02	–					
5. ESMU	3.59	0.69	–0.02	0.04	0.00	0.11	0.84				
6. LMX	3.74	0.67	0.02	–0.03	0.05	–0.07	0.17**	0.80			
7. SFI	3.04	0.98	0.10	–0.02	–0.04	0.05	0.14*	0.06	0.79		
8. UFR	2.78	0.98	–0.04	–0.04	–0.07	–0.09	0.00	0.04	–0.09	–	
9. ECR	3.37	0.99	–0.03	–0.08	–0.09	0.10	0.16*	0.25**	0.23**	0.03	0.85

*p < 0.05, **p < 0.01, reliabilities in diagonal cells, UE, enterprise social media usage experience, ESMU, enterprise social media usage, LMX, leader member exchange, SFI, support for innovation, UFR, enterprise social media usage frequency, ECR, employee creativity.

For hypotheses testing, first, Hypothesis 1 proposed that enterprise social media usage is positively related to employee creativity via LMX. Results in Table 2, based on a bootstrapping technique using 10,000 bootstrap sample indicate that there is a significant indirect of enterprise social media usage with employee creativity via LMX (Effect = 0.06, *SE* = 0.04, at 95% LLCI = 0.0038, ULCI = 0.1500). The hypothesis predicted that SFI mediates the relationship between enterprise social media usage and employee creativity. Accordingly, the results indicate that there is significant indirect effect of enterprise social media usage on employee creativity via SFI (Effect = 0.04, *SE* = 0.02, at 95% LLCI = 0.0007, ULCI = 0.0796). Together, the empirical analysis provides support for the mediation hypotheses of this study.

**TABLE 2:** Indirect effect of enterprise social media usage on employee creativity via LMX and SFI.

Mediator	Independent variable	Dependent variable	Effect	SE	LLCI	ULCI
Total effect	EMSU	ECR	0.10	0.04	0.0311	0.1884
LMX	EMSU	ECR	0.06	0.04	0.0038	0.1500
SFI	EMSU	ECR	0.04	0.02	0.0007	0.0796

*N = 238; ESMU, enterprise social media usage; LMX, leader member exchange; SFI, support for innovation; ECR, employee creativity; SE, standard error; LLCI, lower limit at 95% confidence interval; ULCI, upper limit at 95% confidence interval; bootstrap sample size 10,000.*

Next, we tested the moderated mediation hypotheses of our model. Hypothesis 3 predicted that enterprise social media usage frequency will moderate the indirect relationship between enterprise social media usage and employee creativity via LMX such that the indirect relationship becomes weak when leaders more frequently use enterprise social media. As hypothesized, the results in Table 3 shows that the indirect effect of enterprise social media usage on employee creativity via LMX is strongest when frequency to use enterprise social media is low (Effect = 0.11, *SE* = 0.05, at 95% LLCI = 0.0306, ULCL 0.2054), then when frequency to use enterprise social media is at mean level (Effect = 0.06, *SE* = 0.04, at 95% LLCI = 0.0056, ULCL 0.1515) and at high level (Effect = 0.02, *SE* = 0.05, at 95% LLCI = –0.068, ULCL 0.1372). Thus, the results support Hypothesis 3.

**TABLE 3:** Conditional indirect effect of enterprise social media usage on employee creativity via LMX and SFI at level of enterprise social media usage frequency.

Moderator	Mediator	Independent variable	Dependent variable	Moderator value	Effect	SE	LLCI	ULCI
UFR	LMX	EMSU	ECR	–0.98	0.11	0.05	0.0306	0.2054
UFR	LMX	EMSU	ECR	0.00	0.06	0.04	0.0056	0.1515
UFR	LMX	EMSU	ECR	0.98	0.02	0.05	–0.068	0.1372
UFR	SFI	EMSU	ECR	–0.98	0.02	0.03	–0.0355	0.0872
UFR	SFI	EMSU	ECR	0.00	0.04	0.02	0.0030	0.0836
UFR	SFI	EMSU	ECR	0.98	0.06	0.03	0.0091	0.1120

*N = 238; ESMU, enterprise social media usage; LMX, leader member exchange; SFI, support for innovation; UFR, enterprise social media usage frequency; ECR, employee creativity; SE, standard error; LLCI, lower limit at 95% confidence interval; ULCI, upper limit at 95% confidence interval; bootstrap sample size 10,000.*

Hypothesis 4 predicted that enterprise social media usage frequency will moderate the indirect relationship between enterprise social media usage and employee creativity via SFI such that the indirect relationship becomes weak when employees more frequently use enterprise social media. Contrary to the proposed hypothesis, our results indicate that indirect effect of enterprise social media usage on employee creativity is stronger when employees more frequently use enterprise social media to interact and communicate with colleagues (Effect = 0.02, *SE* = 0.03, at 95% LLCI = –0.0355, ULCL 0.0872) than when the usage frequency is at mean level (Effect = 0.04, *SE* = 0.02, at 95% LLCI = 0.0003, ULCL 0.0836) or low level (Effect = 0.06, *SE* = 0.03, at 95% LLCI = 0.0091, ULCL 0.112). Thus, the results do not support hypothesis 4.

## Discussion and Implications

### Theoretical Contributions

This study makes several important contributions to the existing research. First, the model of this study integrates social media and organizational behavior to provide a comprehensive theoretical explanation regarding how enterprise social media is linked with employee creativity. In doing so, we identify LMX and SFI as essential mechanisms using social exchange theory that link enterprise social media usage with employee creativity. Thus, this study theoretically elaborates and empirically tests how enterprise social media usage is associated with employee creativity through LMX and SFI.

Second, this study further extends the existing theory related to enterprise social media usage by theorizing that frequency of using enterprise social media is an important boundary condition that affects the indirect relationship between enterprise social media usage and employee creativity via LMX and SFI. On one hand, we found that enterprise social media usage frequency can reduce the strength of the positive indirect effect of enterprise social media usage on employee creativity via LMX. This suggests that though enterprise social media usage facilitates the social exchange process among employees and leaders of an organization, yet, more frequent use of enterprise social media can negatively impact the quality of these exchange processes (LMX) and related employee creativity. On the other hand, interestingly, we found that when employees more frequently use enterprise social media to interact and communicate with their co-workers, this strengthens the indirect effect of enterprise social media usage on employee creativity via SFI. This brings an interesting insight into the impact of enterprise social media usage on social exchange relationships and employee creativity. We invite future scholars to shed light on the differentiated nature of exchange relationships and suggest scholars investigate other antecedents of exchange relationships.

Finally, this research adds to our knowledge of the impact of enterprise social media usage. More broadly, this study answers the calls to understand how the use of enterprise social media might affect employee creativity through alternative theoretical perspectives (Sigala and Chalkiti, 2015; Wang et al., 2021). This study based on the social exchange theory, provides a framework to integrate enterprise social media usage, social exchange relationships, and enterprise social media usage frequency, to understand employee creativity. Thus, our moderated mediation model further contributes to our understanding of the mechanisms and implications of enterprise social media adoption by organizations.

### Practical Implications

In this study, we found that enterprise social media usage has significant implications for employee creativity. These findings provide important implications for the managers. Managers who want their employees to generate creativity should adopt social media technologies to facilitate their interaction and communication. Especially, in the current pandemic situation, the use of enterprise social media is found to have a valuable impact on employees’ creativity. In doing so, managers should encourage subordinates to share their ideas and knowledge. For example, managers can assign group tasks to the employees to increase their interaction and promote exchange relationships among them.

Second, we found that the indirect impact enterprise social media usage has on employee creativity via LMX would be weaker when employees more frequently use enterprise social media. This suggests that solely relying on enterprise social media for interaction, communication, and exchange of knowledge and idea with leaders has negative implications for employees’ creativity. As such, though, organizations adopt social technologies to facilitate employees and enhance their creativity and task performance (Wei et al., 2020), yet, employees need to control their use of social technologies, and should also have physical interaction and meetings with leaders. Physical interaction may enhance their quality of exchange relationships resulting in higher creativity. Yet, on the other interestingly, frequent social media usage has strengthened the impact of enterprise social media usage on employee creativity via SFI. This indicates that exchange relationships among colleagues when supported by frequent social media users create a sense that employees support ideas, and support each other to produce more creativity. Thus, managers are suggested to develop an integrated work system in which employees use social technologies and also have physical interaction and meetings. This will create a better understanding of the skills and knowledge and create a collaborative workplace where creativity is supported (Ali et al., 2019). This integration may facilitate the type of interactions that are supportive of creativity.

Finally, as the workplace is facing a transformation from a physical workplace to virtual workstations due to the current outbreak of the Covid19 pandemic, the nature of the workplace has changed. In the current workplace, employees are more focusing on the use of technologies to perform their jobs. This requires managers to design social technologies which could revolve around changing demands of the workplace and facilitate social exchange processes and employee creativity.

### Limitations

This study is not without limitations which highlight the need for future research. First, all of the samples were collected from developing region, which might limit the generalizability of the findings. Research has long realized that cultures vary among countries that might have a significant impact on how employees interact and perform their tasks (Bahadur et al., 2019; Ali et al., 2021b; Islam et al., 2021). Thus, our conclusion regarding the role of enterprise social media in enhancing employee creativity via LMX and SFI might not be generalizable to other cultural settings. Therefore, we invite scholars to investigate our model in Western culture for the generalizability of our findings.

Second, though, we used time-lagged data set in which the outcome variable is measured at the second phase of the survey. However, all the variables are self-reported which generates the possibility of common method bias. To further strengthen the results of this study, we invite future studies to use objective data regarding employee creativity (i.e., creativity rewards) to generate robust results.

Finally, future research should continue to extend the nomological network of social media usage. For example, although, we found that enterprise social media usage is positively related to employee creativity mediated by LMX and SFI, there is a negative impact of usage frequency on the indirect relationship between enterprise social media usage and employee creativity via LMX and SFI. This highlights the possibility that enterprise social media usage is not always positive, indeed in line with Yu et al. (2018) we found that more frequent use of enterprise social media has negative consequences. Yet, research is limited on to what degree enterprise social media usage is effective for employee creativity. This identifies that future research should use the diary method to understand when and to what degree use of enterprise social media might be beneficial to the employees.

## Conclusion

In testing the theoretical model delineating the potential influence of enterprise social media on employee creativity. This study has been able to theoretically elaborate using social exchange theory that LMX and SFI are important mechanisms that transfer the influence of enterprise social media usage to employee creativity. Furthermore, empirical findings indicate that social media usage frequency by employees has a distinct effect on employee creativity via LMX and SFI. Thus, our findings set the stage for further research in understanding how enterprise social media usage by employees and leaders is affecting employees’ creativity.

## Data Availability Statement

The raw data supporting the conclusions of this article will be made available by the authors, without undue reservation.

## Author Contributions

ZW and MH: conception and design of study. AA: acquisition of data. ZW: analysis and interpreting data. ZW, AA, MH, and MG: drafting the manuscript. All authors made substantial contributions to the work reported in the manuscript.

## Conflict of Interest

The authors declare that the research was conducted in the absence of any commercial or financial relationships that could be construed as a potential conflict of interest.

## Publisher’s Note

All claims expressed in this article are solely those of the authors and do not necessarily represent those of their affiliated organizations, or those of the publisher, the editors and the reviewers. Any product that may be evaluated in this article, or claim that may be made by its manufacturer, is not guaranteed or endorsed by the publisher.
